# Ruptured Sinus of Valsalva Aneurysm Accompanied by Aortic and Tricuspid Valve Endocarditis: A Case Report

**Published:** 2017-01

**Authors:** Tahereh Davarpasand, Ali Hosseinsabet, Kyomars Abbasi

**Affiliations:** *Tehran Heart Center, Tehran University of Medical Sciences, Tehran, Iran.*

**Keywords:** *Endocarditis*, *Sinus of Valsalva*, *Aortic valve*, *Tricuspid valve*

## Abstract

A ruptured sinus of Valsalva aneurysm rarely accompanies the aortic and tricuspid valve endocarditis. A 36-year-old woman presented with low-threshold dyspnea on exertion and fever. Transthoracic and transesophageal echocardiography showed a ruptured noncoronary sinus of Valsalva aneurysm with large vegetations on the tricuspid and aortic valves, resulting in moderately severe tricuspid regurgitation and severe aortic regurgitation. Blood culture was negative. The patient was initially treated with antibiotics and then subjected to the surgical repair of the sinus of Valsalva aneurysm and the tricuspid and aortic valve replacement. The patient's postoperative period was uneventful, and she was discharged healthy.

## Introduction

The sinus of Valsalva aneurysm (SVA) is defined as a significant dilatation of the aortic wall between the aortic valve and the sinotubular junction.^1^ The right coronary sinus is the most frequently affected Valsalva sinus, followed by the noncoronary sinus and rarely, the left coronary sinus.^2^^-^^4^ Furthermore, the most common complication of the SVA is its rupture into the right ventricle or atrium and rarely towards the left chambers.^2^^-^^5^ Endocarditis may occur as a complication of the ruptured SVA.^6^

We describe a patient with a ruptured noncoronary SVA associated with aortic and tricuspid endocarditis, which was misdiagnosed initially as a ventricular septal defect with pneumonia and pulmonary thromboemboli.

## Case Report

A 36-year-old woman referred to the emergency department with complaints of fever, dyspnea on exertion (functional class III), pleuritic chest pain of 30 days’ duration, and increased breathlessness since 10 days before admission to our hospital. Her past history revealed complaints of exertional dyspnea of 5 years’ duration. Primary investigation via transthoracic echocardiography led to the false diagnosis of a perimembranous ventricular septal defect, for which the patient was placed under outpatient follow-up. Ten days after the beginning of the new symptoms, she was admitted to a general hospital, where lung spiral computed tomographic scan revealed ill-defined and wedge-shaped subpleural consolidations in the right lung. Consequently, antibiotics (ceftriaxone, ciprofloxacin, and azithromycin) for the treatment of pneumonia and warfarin for the treatment of pulmonary thromboemboli were commenced. 

On the first clinical examinations in our center, the patient was febrile (38.7 ^°^C) and had a regular pulse rate of 110 beats per minute and blood pressure of 100/60 mmHg. The cardiac apex was hyperdynamic. Heart auscultation revealed a continuous murmur at the left sternal border, and lung auscultation showed basilar crackles in the right lung. The spleen was not palpable. There was no lower extremity edema or purpura. The remainder of her physical examinations was unremarkable. The electrocardiogram illustrated sinus tachycardia with left axis deviation. Laboratory investigations showed hemoglobin of 11.62 gr/dL, total leukocyte count of 11 510/mm^3^ with 80.9% neutrophils, platelet count of 265 000/mm^3^, normal serum blood sugar and electrolytes, serum urea of 10 mg/dL, creatinine of 0.6 mg/dL, erythrocyte sedimentation rate of 25 mm/h and C-reactive protein of 8.11 mg/dL. Three sets of blood culture, including anaerobic culture, were drawn 0.5 hour apart, and they revealed no organism growth after 14 days’ incubation period. On the first chest X-ray, the ground glass appearance was seen in the lower lobe of the right lung. 

**Figure 1:**
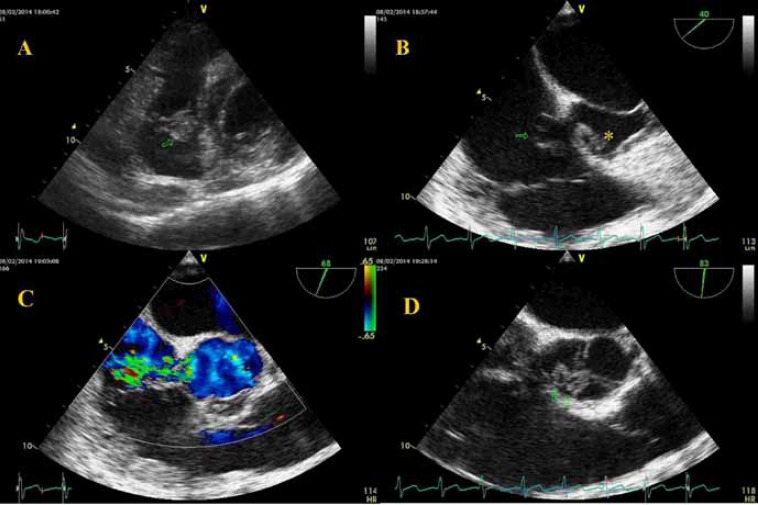
Echocardiographic views of the patient:

Transthoracic and transesophageal echocardiography revealed a ruptured noncoronary SVA to the right atrium, 2 vegetations on the ventricular side of the noncoronary cusp of the aortic valve (7 × 6 mm, 3 × 3 mm) and perforation of this cusp resulting in severe aortic regurgitation, 2 large vegetations on the atrial side of the septal and posterior leaflets of the tricuspid valve (7 × 12 mm, 7 × 12 mm), and moderately severe tricuspid regurgitation (Figure 1). Also, there was a small secundum type atrial septal defect (1.5 mm). The left ventricle was enlarged with mild systolic dysfunction (ejection fraction = 50%), and the right ventricle was enlarged with significant systolic dysfunction. Both atria were enlarged, and the estimated systolic pulmonary artery pressure was 50 mmHg. 

The echocardiographic findings denoted a partially treated infective endocarditis. Accordingly, an empirical intravenous antibiotics regimen for negative-culture endocarditis was started, and the patient was prepared for surgery after 10 days’ antibiotics therapy. After aorto-bicaval total cardiopulmonary bypass, the rupture of the SVA was repaired with a double patch, the aortic valve was replaced with a mechanical prosthetic valve (S. J. Regent, 23 mm), and the tricuspid valve was replaced with a bioprosthetic valve (Hancock, 31 mm). The extent of the damage to the aortic and tricuspid valves and the extension of the infection of the subvalvular apparatus of the tricuspid valve rendered repair impossible. However, the small atrial septal defect was sutured. Postoperative transthoracic echocardiography showed no residual shunts within the reconstructed noncoronary sinus. In addition, there was a normal function of the aortic and tricuspid prosthetic valves with no paravalvular leakage. The estimated systolic pulmonary artery pressure was 32 mmHg. Special stains of the vegetations did not show any bacteria. Postoperative antibiotics were continued for 4 weeks, and the patient had an uneventful recovery period. 

A) Transthoracic echocardiography in the parasternal short-axis view shows tricuspid valve vegetation (head arrow).

B) Transesophageal echocardiography (40^°^) shows a ruptured noncoronary cusp to the right atrium (arrow) and the aortic valve vegetation (*).

C: Transesophageal echocardiography (68^°^) shows a left-to-right shunt from the ruptured sinus of Valsalva aneurysm.

D) Transesophageal echocardiography (83^°^) shows a cross-section of the aortic valve vegetations and perforations (arrows).

AV, Aortic valve; LA, Left atrium; LV, Left ventricle; RA, Right atrium; RV, Right ventricle

***To watch the following videos, please refer to the relevant URLs:***

Video 1. Large vegetation on the tricuspid valve

http://jthc.tums.ac.ir/index.php/jthc/article/view/671/506

Video 2. Aneurysm of the noncoronary sinus of Valsalva to the right atrium

http://jthc.tums.ac.ir/index.php/jthc/article/view/671/507

Video 3. Ruptured aneurysm of the noncoronary sinus of Valsalva to the right atrium with a left-to-right shunt

http://jthc.tums.ac.ir/index.php/jthc/article/view/671/508

Video 4. Vegetation and perforation of the aortic valve

http://jthc.tums.ac.ir/index.php/jthc/article/view/671/509

## Discussion

The majority of ruptured SVAs are congenital due to a defect in the aortic media; nevertheless, they can occasionally be secondary.^5^^, ^^7^ Patients with small perforations of aneurysms always have an asymptomatic murmur, but they may present with symptoms of infective endocarditis.^7^^, ^^8^ There are reports of right-sided endocarditis, tricuspid valve^9^^-^^11^ or pulmonic valve^12^^-^^13^ accompanied by ruptured SVA, isolated or accompanied by aortic valve endocarditis.^14^^, ^^15^

The literature contains only a few reports on the coexistence of the aortic valve and tricuspid valve endocarditis. Jung et al.^15^ reported a patient with a ruptured noncoronary SVA to the right atrium, accompanied by the tricuspid (anterior and septal leaflets) and aortic valve vegetations. The severity of the aortic valve regurgitation was trivial, and the severity of the tricuspid valve regurgitation was moderate. In blood culture, *Enterococcus gallinarum* grew. In our patient, although there was a similar congenital defect, the posterior and septal leaflets of the tricuspid valve were involved and there was severe aortic and moderately severe tricuspid valve regurgitation. Also, blood cultures were negative, and the clinical course was different. Elsewhere Lin et al.^14^ reported a similar case but with vegetations seen in the operating room and not on echocardiography only in the septal leaflet of the tricuspid and aortic valves The authors did not report any regurgitation of the tricuspid and aortic valves, and their patient's blood culture showed *Streptococcus viridans*. Also, the clinical course of their patient was different from that of ours. Therefore, we present the first report on a patient with a ruptured noncoronary SVA aneurysm, together with the tricuspid (septal and posterior leaflets) and aortic valve culture-negative endocarditis and severe aortic and moderately severe tricuspid valve regurgitation. In all probability, given our patient's past medical history, which included the misdiagnosis of this congenital defect, a left-to-right shunt flow may have predisposed her to endocarditis of the aortic and tricuspid valves. The wedge-shaped lesions in the right lung may have been due to the embolization of the vegetations. In our patient, the aortic and tricuspid valve repair was impossible owing to the extensive involvement of the valves. She underwent the aortic and tricuspid valve replacement with the repair of the rupture of the SVA. Surgical repair for the ruptured SVA has low operative mortality and high long-term survival rates. It is preferable to preserve the aortic valve; however, the aortic valve replacement may be necessary in significant aortic regurgitation.^5^

## Conclusion

Although endocarditis does not commonly accompany a ruptured SVA, its existence should be kept in mind when evaluating or indeed when operating on a patient with that condition. Therefore, all the heart valves should be explored for the evidence and complications of endocarditis. 

## Notes:

***This paper should be cited as: ***Davarpasand T, Hosseinsabet A, Abbasi K. Ruptured Sinus of Valsalva Aneurysm Accompanied by Aortic and Tricuspid Valve Endocarditis. J Teh Univ Heart Ctr 2017;12(1):42-45.
